# Vascular emergencies in neuro-ophthalmology

**DOI:** 10.22336/rjo.2020.54

**Published:** 2020

**Authors:** Eugenia Raluca Iorga, Dănuț Costin

**Affiliations:** * Department of Ophthalmology, “N. Oblu” Clinical Emergency Hospital, Iași, Romania; ** Department of Ophthalmology, “Gr. T. Popa” University of Medicine, Iași, Romania

**Keywords:** carotid artery stenosis, ocular ischemic syndrome, transient monocular blindness, oculomotor palsies, Claude Bernard Horner syndrome

## Abstract

The cerebral vascularization is assured by the 2 internal carotids and 2 vertebral arteries, and the Willis circle.

Carotid artery obstruction is the most common abnormality associated with ocular ischemic syndrome. Obstruction may be due to atheromatous plaque, external compression, arteritis, or dissection of the artery. An atheromatous lesion of the carotid artery is the most frequent lesion responsible for ocular ischemic syndrome. The signs and symptoms of ocular ischemic syndrome are associated with severe hypoperfusion of the eye. Inflammatory lesions of the carotid artery are responsible for decreased flow in the carotid system. Other vascular emergencies are carotid artery dissection, Horton arteritis, aneurysms and carotid-cavernous fistula.

The most common ocular signs and symptoms are transient monocular blindness, persistent monocular blindness, ocular ischemia, Claude Bernard Horner syndrome and oculomotor palsies.

The carotid pathology can be a life-threatening pathology and it is important to recognize all these signs and symptoms. A multi-specialty approach will prevent misdiagnosis and lead to a better patient management.

**Abbreviations:** OIS = ocular ischemic syndrome, TMB = transient monocular blindness, TIA = transient ischemic attack, ESR = erythrocyte sedimentation rate, CRP = C reactive protein, NVE = neovascularization elsewhere in the retina, NVD = neovascularization on the disc, AION A = anterior ischemic arteritic optic neuropathy, CBH = Claude Bernard Horner syndrome, MRI = magnetic resonance imaging

## Introduction

**Fig. 1:**
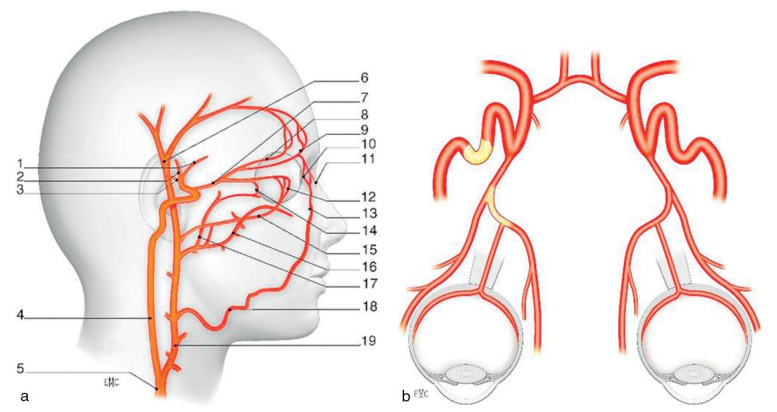
Cerebral and ocular vascularization. a. Cerebral vascularization. 1: anterior cerebral artery; 2: middle cerebral artery; 3: anterior communicating artery; 4: artery internal carotid; 5: common carotid artery; 6: superficial temporal artery; 7: ophthalmic artery; 8: supraorbital artery; 9: artery supratrochlear; 10: median palpebral artery; 11: dorsal artery of the nose; 12: lateral palpebral artery; 13: angular artery; 14: artery lacrimal; 15: transverse facial artery; 16: maxillary artery; 17: middle meningeal artery; 18: facial artery; 19: external carotid artery. b. Vascularization of the eyeballs by ophthalmic artery, which is a branch of the internal carotid artery. (from Vignal-Clermont C, Tilikete C, Milea D. Neuro-ophtalmologie. 2e edition, 2016, Elsevier)

The cerebral vascularization is assured by internal carotid arteries, vertebral arteries and the Willis circle. The common carotid arteries split into the external and internal carotid arteries at the upper border of the thyroid cartilage. The internal carotid takes a deeper path, it supplies the cerebral vascularization. The external carotid artery vascularizes the neck and the face [**1**,**2**], (**Fig. 1**).

Carotid pathology and the eye: the carotid pathology gives ophthalmological, monocular, ipsilateral symptoms. Carotid damage involves a double risk-blindness and stroke. Among the carotid lesions that can give ophthalmic symptoms, the most frequent are the carotid stenosis and carotid occlusions, and the malformations-aneurysms and carotid-cavernous fistula. The carotid occlusive disease can be caused by atheroma, dissections and trauma, fibromuscular dysplasia, Takayasu's arteritis, external compression, emboli from heart, inflammatory and infectious disorders.

The ocular ischemic syndrome (OIS) is seen in severe carotid occlusive disease, because this pathology is associated with severe hypoperfusion of the eye. For this syndrome to appear, it is necessary to have a stenosis of the ipsilateral carotid artery of 90% or greater [**3**].

 The most important cerebral vascular emergencies with ophthalmic implications are severe carotid stenosis, carotid artery dissection, Horton arteritis, rupture of an aneurysm and carotid-cavernous fistula [**4**].

1. Carotid stenosis

**Fig. 2:**
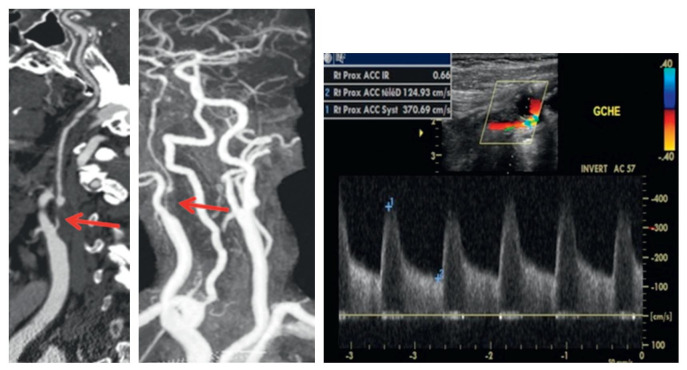
Angio-scanner (a) and MRA (b) of the supra-aortic trunks, carotid Doppler (c). Tight right carotid stenosis (from Vignal-Clermont C, Tilikete C, Milea D. Neuro-ophtalmologie. 2e edition, 2016, Elsevier)

The patients with carotid stenosis can present with:

• pain;

• transient monocular blindness (TMB);

• persistent monocular blindness - Central retinal artery occlusion, ophthalmic artery occlusion, ischemic optic neuropathy; 

• oculomotor paralysis; 

• venous stasis and ocular ischemic syndrome;

• Claude Bernard Horner syndrome; 

• asymptomatic retinal embolism [**4**-**6**], (**Fig. 2**).

Mizener et al. investigated 39 eyes of 32 patients with OIS, and noticed that 41% of the eyes had sudden vision loss, 15% amaurosis fugax and 13% eyes had eye or orbital pain [**7**].

The most encountered symptom in carotid occlusive disease is the transient monocular blindness or *amaurosis fugax* (TMB). The patient complains of a painless, sudden monocular visual loss that lasts between 2 to 30 minutes. After this episode, the vision recovers completely. The eye exam in-between the episodes is usually normal. Fisher et al. reported the association of amaurosis fugax with contralateral hemiplegia in patients with severe internal carotid artery occlusion [**8**,**9**]. Regarding the physiopathology of the transient monocular blindness, two mechanisms may be responsible - first, an embolic mechanism (fibrin-platelet emboli, cholesterol emboli and calcium emboli) that causes stenosis, and second, a hemodynamic mechanism, in which authors describe longer TMB, as it can be seen in severe carotid stenosis [**9**-**11**].

The severe carotid stenosis carries a triple risk: of blindness, ischemic stroke and myocardial infarction [**11**,**12**].

**Fig. 3:**
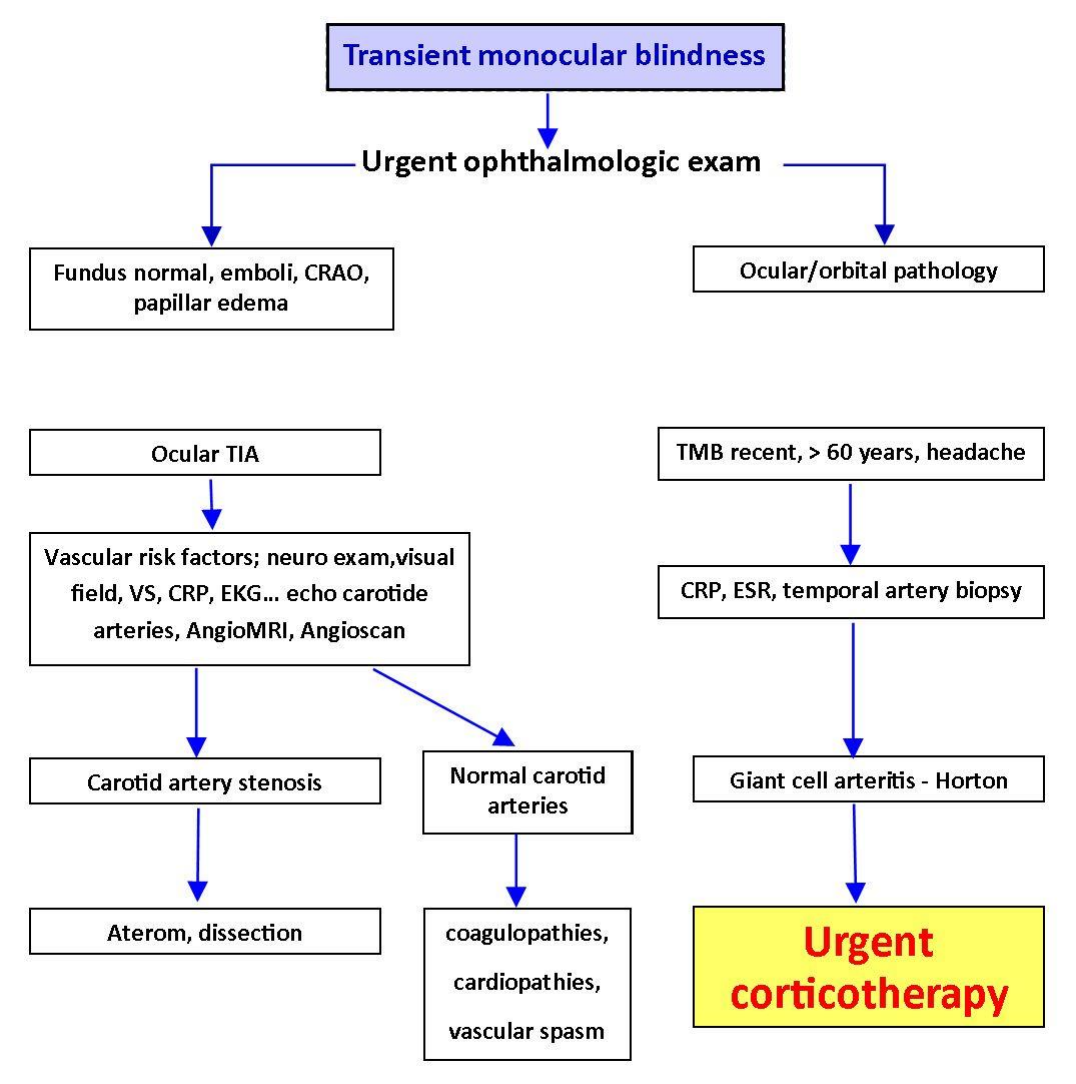
Management of the transient monocular blindness

A patient with TMB needs an urgent exam. This must include an ophthalmological examination, a general clinical examination, a neurological exam, a cardiovascular exam and other specific investigations, such as Doppler ultrasound, inflammatory markers (erythrocyte sedimentation rate ESR, reactive protein C CRP), EKG, AngioMRI or angioscan. The fundus exam may often be normal, but sometimes it can show retinal emboli, and even retinal arterial occlusion. The general exam should include palpation of the temporal arteries, in order to detect Horton arteritis. The neurological exam aims to identify a hemispheric transient ischemic attack (TIA) [**13**], (**Fig. 3**).

*Persistent monocular blindness* is less common encountered and it can have different causes. The most common is the central retinal artery occlusion that can be seen in tight carotid stenosis. The patient complains of sudden loss of visual acuity. Another cause, much rarer, is ophthalmic artery occlusion. This can cause ischemia of the retina, the choroid and the optic nerve (anterior ischemic optic neuropathy). The patient presents with a sudden decrease in visual acuity and the fundus exam can show papillary edema and retinal neovascularization [**13**].

*The stasis retinopathy* is characterized by dilated retinal veins, peripheral microaneurysms and flame-shaped hemorrhages in the peripheral retina and narrow retinal arteries. These are the most frequent changes in carotid obstructive disease, as a result of chronic ischemia [**13**-**15**].

Chronic ocular hypoperfusion, characteristic in carotid occlusive disease, causes *ocular ischemic syndrome*. Severe occlusion of the carotid artery induces hypoperfusion into the ophthalmic artery, which precipitates ocular ischemia. At a slit-lamp exam of the anterior pole, one can observe neovascularization of the iris-rubeosis iridis. Rubeosis iridis can develop in eyes with central retinal vein occlusion, but also in patients with carotid artery obstruction [**16**]. It can be seen in patients with ocular ischemic syndrome, at the time of presentation, in two-thirds of the eyes [**17**]. Neovascularization can also be seen in the posterior segment, on the optic disc or in the retina [**18**]. Mizener reported the presence of rubeosis iridis in 34 of 39 eyes with OIS at the time of the initial visit [**7**]. Sivalingam and colleagues reported the association of systemic hypertension and diabetes in 73%, and respectively 56% of the patients with ocular ischemia [**19**]. 

Whenever carotid artery disease is suspected, a prompt noninvasive exam is necessary to confirm the carotid disease. It is also important to determine its cause (atheroma, dissection, vasculitis, compression) and to assess the severity of the lesion. The most common exams are cerebral angiography, echo Doppler, magnetic resonance angiography and spiral CT angiography [**20**-**24**]. 

The treatment of ischemic eye syndrome is often controversial. The management of the ocular ischemic syndrome is aimed to treat the iris neovascularization and the neovascular glaucoma. The treatment of neovascular glaucoma includes panretinal photocoagulation laser, trabeculectomy with antimetabolites and, for more severe cases, drainage shunts. The treatment of OIS also consists of anticoagulant drugs, endarterectomy, in order to restore the blood flow. Aspirin is frequently given to these patients, as it inhibits platelet aggregation by blocking the cyclooxygenase pathway. A meta-analysis showed a 20-25% reduction in the risk of stroke for the patients treated with aspirin [**25**].

The management of ocular ischemic syndrome is difficult and controversial. Some doctors recommend that patients with isolated transient ocular ischemic attack should be treated with medical therapy, no matter how tight the stenosis is. Other doctors recommend endarterectomy, even in asymptomatic patients [**26**,**27**].

**Fig. 4:**
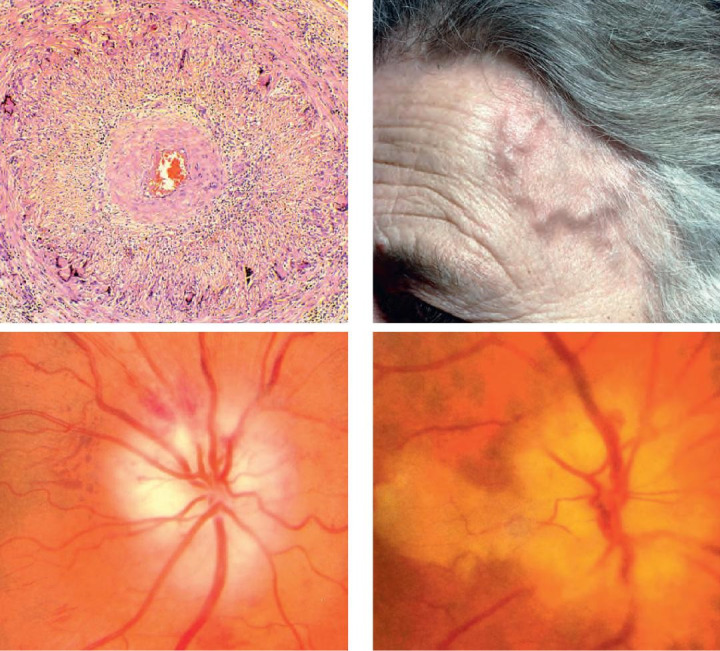
Giant cell arteritis - Horton. A) Histology - granulomatous inflammation and narrowing of the lumen; (B) the superficial temporal artery is pulseless and thickened; (C) pale swollen disc; (D) papillary oedema and cilioretinal artery occlusion (from Bowling B. Kanski’s Clinical Ophthalmology. A systematic approach. Eighth edition, 2016, Elsevier)

2. Horton arteritis - anterior ischemic arteritic optic neuropathy is an ophthalmic emergency that usually occurs in patients over 60 years old. These patients present an altered general condition and complain of headache, significant decrease in visual acuity, claudication on mastication, pseudo polyarthritis and weight loss. The blood tests show very high erythrocyte sedimentation rate ESR and CRP values. On fundus examination, one can detect a pale papillary edema [**13**]. The treatment consists of intravenous bolus of corticosteroids, in order to prevent bilateral vision loss. Secondly, a temporal artery biopsy is performed to confirm the diagnosis (**Fig. 4**).

3. Carotid artery dissection

Carotid artery dissection is usually suspected in young patients with painful Claude Bernard Horner syndrome. Patients present with headache, eyelid ptosis and ipsilateral miosis [**28**].

**Fig. 5:**
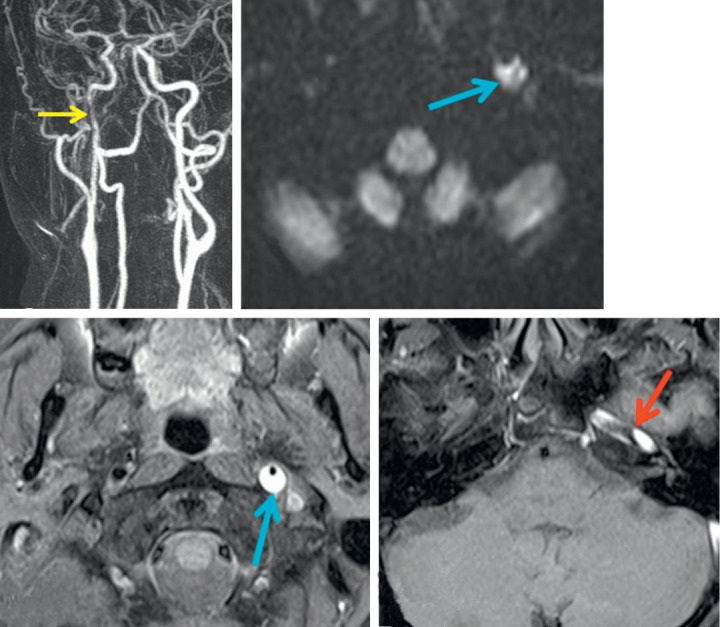
Angio MRI of the supra aortic trunks (a); MRI with diffusion slices B1000 (b); T1 gadolinium FATSAT (c,d); Carotid hematoma (c) with carotid stenosis (a). Carotid dissection with intracranial extension (d) (from Vignal-Clermont C, Tilikete C, Milea D. Neuro-ophtalmologie. 2e edition, 2016, Elsevier)

Studies showed that patients with spontaneous dissection of the internal carotid artery can also have a history of stroke in their family [**29**]. The most frequent cause of carotid artery dissection is severe trauma to the head and neck. Approximately 1% of the patients admitted for serious road accidents suffer a blunt carotid lesion, including intimal dissections, thrombosis or fistulas [**30**], (**Fig. 5**).

**Fig. 6:**
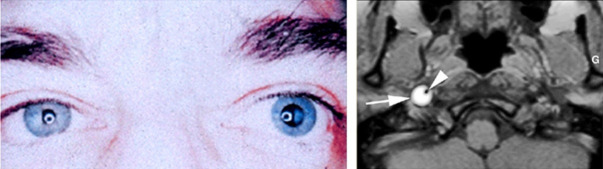
Carotid dissection - painful Claude Bernard Horner (from DU Neuro-ophtalmologie, 2018-2019, Paris, Pupilles, Lamirel)

The signs and symptoms of carotid dissection are: local signs (80%), pain (headache, sore throat), cranial nerve palsies, CBH, TMB and ischemic signs, such as ischemic stroke, TIA, but are also asymptomatic in 10-20% of the cases [**30**], (**Fig. 6**).

The treatment includes observation, anticoagulant medication, implantation of a stent, and carotid artery ligation. 

4. The carotid-cavernous fistula 

The carotid-cavernous fistula is an abnormal communication between the internal carotid artery and the cavernous sinus. The most common types of fistulas are the direct shunts, between the artery and the cavernous sinus. They often occur as a post traumatic lesion. The indirect shunts represent congenital communications between an artery and a vein.

**Fig. 7:**
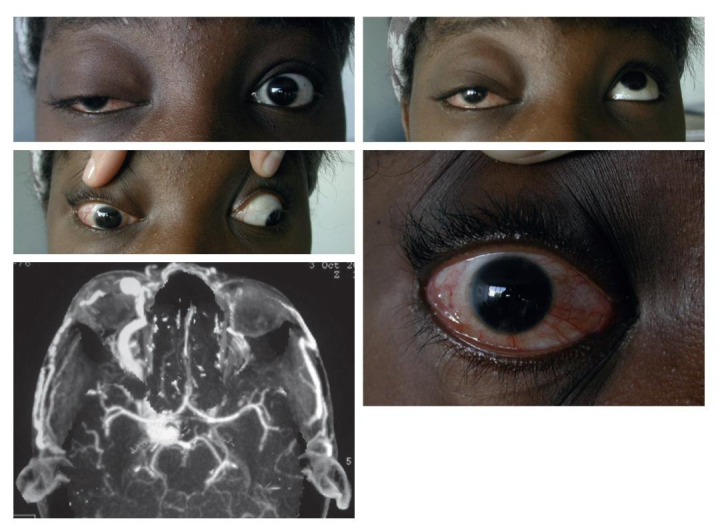
Post-traumatic carotid-cavernous fistula - ptosis, exophthalmos and hypotropia of the eye right (a), global ocular motility disorders (b-d). The episcleral veins are evident (d). Angio-scanner - visualization of the carotid-cavernous fistula and dilation of the ophthalmic vein (e) (from Vignal-Clermont C, Tilikete C, Milea D. Neuro-ophtalmologie. 2e edition, 2016, Elsevier)

The signs and symptoms seen in the direct carotid-cavernous fistula are represented by the triad - pulsatile proptosis + chemosis + intracranial whistling [**4**]. Clinically, there is severe conjunctival congestion, hemorrhagic chemosis, ptosis and pulsatile proptosis accompanied by a whistling. One can encounter painful ophthalmoplegia, the most common being the VI nerve palsy. Fundus examination shows papillary edema, intraretinal hemorrhage and dilated veins. The prominence of the superior ophthalmic vein and the thickening of the extraocular muscles were shown on the CT and MRI. The treatment consists of interventional radiology, such as closure of the fistula with a removable balloon or surgery [**31**], (**Fig. 7**).

The indirect carotid-cavernous fistula has a low flow. Clinically, there is moderate ocular congestion, mild proptosis and ocular pulsation on aplanotonometry. The fundus examination appears normal, but sometimes it may present tortuous veins.

5. Intracranial aneurysms

**Fig. 8:**
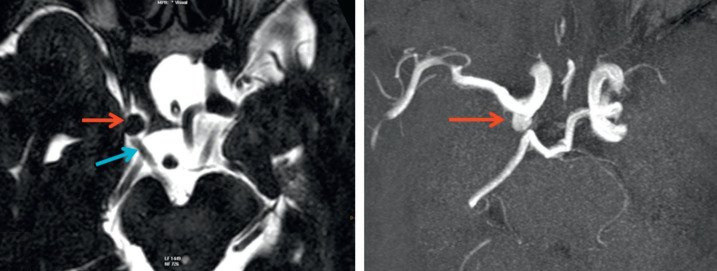
MRI axial T2 (a) and TOF (b). Posterior communicating artery aneurysm compressing the IIIrd oculomotor nerve (from Vignal-Clermont C, Tilikete C, Milea D. Neuro-ophtalmologie. 2e edition, 2016, Elsevier)

The intracranial aneurysms represent acquired ecstasies of an artery, located at the bifurcations of the arteries at the base of the skull. The most common complication of an aneurysm rupture is meningeal hemorrhage, which has a high mortality rate. 12% of the patients die before reaching the hospital, 40% of them die in the first month, and those who survive, have severe complications [**32**]. Most aneurysms are asymptomatic until their rupture, and only a few have symptoms related to compression of the adjacent structures. Headache is the most common symptom. Neuro-ophthalmic symptoms are common and depend on the location of the aneurysm (**Fig. 8**).

**Fig. 9:**
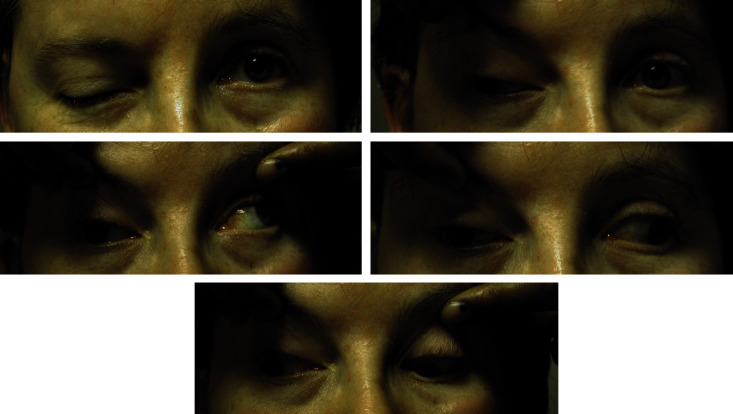
Complete III nerve palsy right eye. - complete right ptosis (a) the eye is in divergence and hypotropia, (b) pupil - mydriasis, (c) impossibility of elevation, (d) adduction and (e) lowering of the globe (from Vignal-Clermont C, Tilikete C, Milea D. Neuro-ophtalmologie. 2e edition, 2016, Elsevier)

The ophthalmic manifestations are multiple: some are the consequence of an aneurysmal rupture, such as IIIrd nerve paralysis, carotid-cavernous fistula, Terson syndrome and some are following compression by a giant aneurysm [**32**], (**Fig. 9**).

The diagnosis is made in the first week by a CT scan and the MRI exam is useful in the first month. It is important that the lumbar puncture is made in the first month [**33**].

The treatment consists of excluding the aneurysm from the intracranial circulation, either by surgery (clip to the neck of the aneurysm) or endovascular treatment (embolization) [**33**]. 

Aneurysms are located at the terminal level of the internal carotid artery, at the origin of the posterior communicating artery. In 90% of the ruptured aneurysms, the IIIrd nerve palsy precedes the rupture [**34**]. Elements in favor of an aneurysmal rupture, easy to remember, are the rule of the 4 P - Pupil - mydriasis, Partial, Progressive, Pain [**4**].

Terson syndrome refers to vitreous hemorrhage in association with subarachnoid hemorrhage. The sudden increase in intracranial pressure causes a sudden increase in venous pressure and rupture of the preretinal capillaries, giving rise to a vitreous hemorrhage [**35**]. Terson syndrome occurs in 10-40% of the aneurysm ruptures [**36**]. The diagnosis is made on fundus examination and ocular ultrasound. 

## Conclusions

The carotid pathology can be a life-threatening pathology and it is important to recognize all these signs and symptoms. A multi-specialty approach will prevent misdiagnosis and lead to better patient management.

**Conflict of interest**

The authors state no conflict of interest.

**Informed Consent**

Informed consent has been obtained from all individuals included in the study.

**Authorization for the use of human subjects**

The research related to human use complies with all the relevant national regulations, institutional policies, is in accordance with the tenets of the Helsinki Declaration, and has been approved by the ethics committee of Department of Ophthalmology, “N. Oblu” Clinical Emergency Hospital, Iași, Romania.

**Acknowledgements**

None.

**Sources of Funding**

None.

**Disclosures**

None.
